# A Series of Anatomical Sketches and Diagrams, with Descriptions and References

**Published:** 1838-07

**Authors:** 


Art. XII.—*A Series of Anatomical Sketches and Diagrams, with Descriptions and References.* By Thomas Wormald, Assistant Surgeon and Demonstrator of Anatomy in St. Bartholomew's Hospital; and M. M'Whinnie, Teacher of Practical Anatomy at St. Bartholomew's Hospital.—*London*, 1838. Part I. 4to. pp. 15.

The design of these sketches and diagrams is explained in a brief prefatory notice, in which the authors remark, that "their experience has taught them how much the studies of their pupils are facilitated, and their memories refreshed by sketches taken from nature, or by diagrams made extemporaneously." The objects proposed are meritorious, and the execution of the sketches which compose the present number are remarkable for their correctness, perspicuity, and neatness of execution. We do not, however, quite coincide with the authors in believing that they will *permanently* benefit the student by assistance like the present. Our own experience in these matters disinclines us to favour the system of rendering the acquirement of anatomy too simple, or of affording assistance which may in any way tend to diminish the necessity of actual dissection. The retention, rather than the easy attainment of practical information, ought to be the ulterior object to be constantly kept in view. This remark is more or less applicable to the study of our profession generally, and indeed to the acquirement of every sort of information. Where knowledge is presented to the enquirer, as it were ready-made, or by wholesale, he rarely either appreciates it so thoroughly or retains it so long, as if the gaining of it had cost him more time and labour; and certainly the intellect loses that wholesome exercise which ought to be a main object in all mental training. We have ourselves found very considerable advantage in extemporaneous sketches for the purpose of illustration; and would greatly encourage the practice of copying such into their note-books, by the students: but this latter practice we regard as one of the principal objects to be attained by their employment. All the assistance of this kind required by the pupil, may be derived from large and boldly painted diagrams of regions, the relative anatomy of which is important in a surgical point of view. If the undertaking of Messrs. W. and M. should grow into a more systematic form as it increases in bulk, we feel satisfied that they will contribute a very valuable work of *reference* to the practitioner as well as the student.

